# Estimation of tumor coverage after RF ablation of hepatocellular carcinoma using single 2D image slices

**DOI:** 10.1007/s11548-025-03423-z

**Published:** 2025-06-07

**Authors:** Nicole Varble, Ming Li, Laetitia Saccenti, Tabea Borde, Antonio Arrichiello, Anna Christou, Katerina Lee, Lindsey Hazen, Sheng Xu, Riccardo Lencioni, Bradford J. Wood

**Affiliations:** 1 Center for Interventional Oncology, National Institutes of Health, Bethesda, MD USA; 2 Philips Healthcare, Cambridge, MA USA; 3 Henri Mondor Biomedical Research Institute, Créteil, France; 4 Department of Diagnostic and Interventional Radiology, UOS of Interventional Radiology, Ospedale Maggiore Di Lodi, Lodi, Italy; 5 Academic Division and School of Radiology, Department of Translational Research, University of Pisa, Pisa, Italy; 6 Radiology & Imaging Sciences/Clinical Center, National Institutes of Health, MSC 1182, Bldg. 10, Room 1C341, Bethesda, MD 20892-1182 USA; 7 National Institute of Biomedical Imaging and Bioengineering, National Institutes of Health, Bethesda, MD USA

**Keywords:** Radiofrequency ablation, Ablation confirmation, AI, HCC

## Abstract

**Purpose:**

To assess the technical success of radiofrequency ablation (RFA) in patients with hepatocellular carcinoma (HCC), an artificial intelligence (AI) model was developed to estimate the tumor coverage without the need for segmentation or registration tools.

**Methods:**

A secondary retrospective analysis of 550 patients in the multicenter and multinational OPTIMA trial (3–7 cm solidary HCC lesions, randomized to RFA or RFA + LTLD) identified 182 patients with well-defined pre-RFA tumor and 1-month post-RFA devascularized ablation zones on enhanced CT. The ground-truth, or percent tumor coverage, was determined based on the result of semi-automatic 3D tumor and ablation zone segmentation and elastic registration. The isocenter of the tumor and ablation was isolated on 2D axial CT images. Feature extraction was performed, and classification and linear regression models were built. Images were augmented, and 728 image pairs were used for training and testing. The estimated percent tumor coverage using the models was compared to ground-truth. Validation was performed on eight patient cases from a separate institution, where RFA was performed, and pre- and post-ablation images were collected.

**Results:**

In testing cohorts, the best model accuracy was with classification and moderate data augmentation (AUC = 0.86, TPR = 0.59, and TNR = 0.89, accuracy = 69%) and regression with random forest (RMSE = 12.6%, MAE = 9.8%). Validation in a separate institution did not achieve accuracy greater than random estimation. Visual review of training cases suggests that poor tumor coverage may be a result of atypical ablation zone shrinkage 1 month post-RFA, which may not be reflected in clinical utilization.

**Conclusion:**

An AI model that uses 2D images at the center of the tumor and 1 month post-ablation can accurately estimate ablation tumor coverage. In separate validation cohorts, translation could be challenging.

## Introduction

Hepatocellular carcinoma (HCC) is the most common type of primary liver cancer, which ranks fourth in most cancer-related deaths worldwide [1]. Percutaneous thermal ablation of liver metastasis has become a standard treatment option early stage or unresectable tumors [2, 3]. Guidelines have stated that image guidance is crucial for all steps of a successful ablation, including planning, targeting, intraprocedural monitoring, and ablation confirmation [4]. The assessment of tumor coverage by the ablation zone is required to minimize disease recurrence. However, it has been shown that simple visual inspection of pre- and post-ablation images is inadequate for true assessment of ablation completeness [5] and may vary dramatically among users regardless of experience level [6].

To improve standardization, three-dimensional imaging software has been proposed and studied to compare tumors to ablated regions [7–10]. This has led to the adoption of minimal ablative margins, or additional treatment boundaries beyond the tumor, which is thought to be highly predictive of local tumor progression [9, 11, 12]. Barriers to widespread clinical adoption of 3D ablation confirmation remain the tools and speed to obtain a result. Image registration techniques (rigid vs. deformable) and ablation margin quantification methodologies can also vary, making translation and standardization challenging [11, 13]. To streamline and standardize this process, some effort has been made to develop AI models for image segmentation or outcome prediction [14–16]. While 3D approaches may offer detailed volumetric information, they often require tedious segmentation and elastic registration techniques that may not be fully validated or integrated into real-time clinical workflows.

Therefore, to provide a rapid estimation of ablation zone coverage, we test the hypothesis that information from an image at the center of the pre-treatment tumor and post-treatment ablation zone can estimate the technical success of the treatment. This study surmises that machine learning and AI models can identify similarities between pre- and post-treatment images to estimate registration quality and overlap of the tumor and ablation zone. This is accomplished by extracting and isolating a single image slice from the center of the region of interest (RIO, tumor or ablation zone) and performing feature extraction to feed into regression and classification models. It was hypothesized that well-aligned tumor and ablation zones would have minimal image feature discrepancy, which could be identified and used to predict tumor coverage. The models are trained and tested as a secondary analysis of an investigation on patients from a multicenter randomized control study of RFA and validated on interventional radiology cases from a separate institution.

## Methods

### Study population

This retrospective study was performed as a secondary analysis on a previously collected prospective, randomized control trial (NCT02112656) [17]. The prospective trial was approved by institutional review board and recruited 556 patients with written informed consent between 2014 and 2018 [17–20]. Patients were > 18 years old and had solitary, non-resectable hepatocellular carcinoma lesions that were ≥ 3 cm and ≤ 7 cm in size. All patients received radiofrequency ablation (RFA), with a minimum dwell time of 45 min, and were randomly assigned to RFA + dummy infusion (RFA only) or RFA + ThermoDox™. Prior analysis showed no different in survival, progression, or patient demographics based on treatment arm, indicating that both treatment groups could be combined [18]. All patients received a pre-treatment contrast-enhanced CT immediately prior to treatment and a post-ablation contrast-enhanced CT 28 days after treatment.

### Generation of ground-truth by segmentation and image registration

To determine the ground-truth of tumor coverage, pre-treatment tumors and post-treatment ablation zones were semi-automatically segmented, and the images were registered. CT images were screened for sufficient image quality for segmentation, which included well-defined tumor and ablation zones as previously described [18]. The arterial phase pre-ablation CT was used for tumor segmentation, and the portal venous phase CT 28 days post-ablation was used for ablation zone segmentation. Images were collected at 5 mm slice thicknesses. The tumor was segmented using a semi-automated threshold-based method with manual corrections by two operators trained and checked by an experienced board-certified radiologist using 3D Slicer (v4.11, http://www.slicer.org) [21]. Segmentations of the ablation zone were performed by two different operators trained and checked by an experienced board-certified radiologist using semi-automatic segmentations with manual corrections (3D Slicer, with NVIDIA annotation _ct_liver_tumor module) and corrected manually.

Registration of the 3D images was performed on matching pairs pre-ablation and 28-day post-ablation CT scans using semi-automatic elastic registration (Elastix) 3D Slicer. Pre-processing of images was performed as previously described [18], and assessment of the registration quality using standard error and structural similarity index was calculated using (MATLAB v.2022b, MathWorks, Natick, MA).

The technical success of the treatment, or the percentage of the tumor covered by the ablation zone (percent tumor coverage), was calculated as the ratio of the tumor volume covered by the ablation divided by the total volume of the tumor. The ground-truth was measured in 3D after elastic registration.

### Data preparation- extraction of image slice and data augmentation

A single image axial slice at the center of the ROI (tumor or ablated tissue) was extracted from both the pre-treatment tumor image and the post-treatment ablation image. The slice was identified by the segmentation, which was created during ground-truth generation described previously. The single slice was then cropped at the center (in coronal-sagittal planes and transversely) to 300 × 300 pixels and padded if necessary to preserve the image size.

To increase the training set size, the images were augmented by the addition of Gaussian noise (non-zero mean with a variance of 0.001) and/or the rotation of the images by 90 degrees.

For model training and testing for each of the models, the cases were randomly split into training and testing cohorts at an 80:20 ratio, respectively. To increase the number of cases and the power of the study, the models were tested in three cohorts: 1. with no data augmentation, 2. with original images plus images with noise added, where images were exclusively paired (i.e., original_tumor_/original_ablation_ or noise_tumor_/noise_ablation_), and 3. with original images and images with noise added, where image pairs were with original or noise could be also be paired with each other (i.e., original_tumor_/noise_ablation_ and noise_tumor_/original_ablation_).

### Feature extraction

**Fig. 1:**
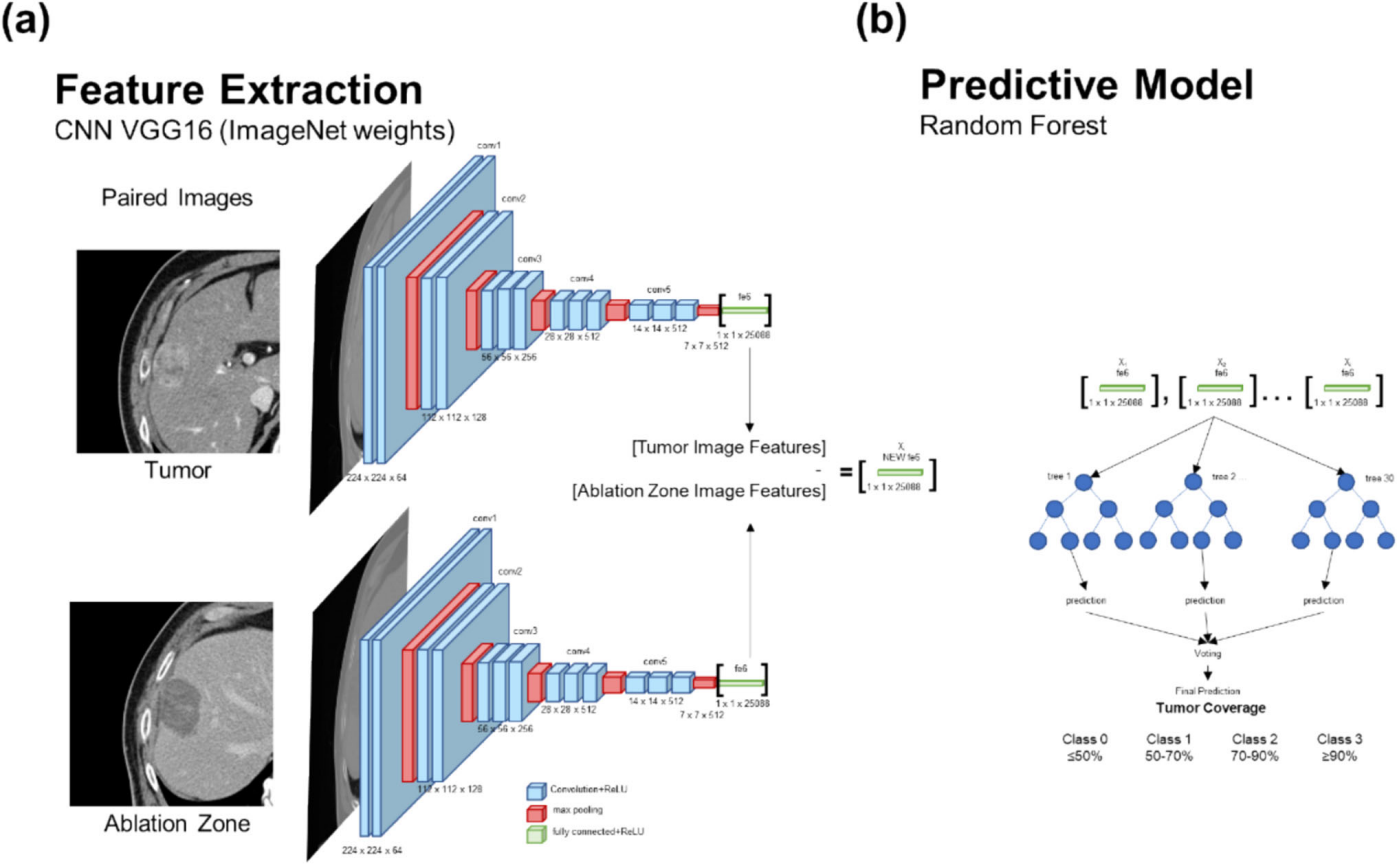
Model architecture that begins with **a** features extraction. Feature extraction is performed using standard ImageNet model weights and placed in a network that generates a single feature set of subtracted features. **b** Classification prediction occurs using a random forest model where features are used to predict the approximate tumor coverage after ablation

To compare the tumor and ablation zone images, features of each image were extracted and compared. The pre-trained VGG16 convolutional neural network was used to extract robust feature representations from input images. Features were extracted up to the fully connected (flattened) layer, yielding a vector of 25,088 features per image. Prior to feature extraction, all input images were preprocessed according to VGG16's requirements, including resizing to 224 × 224 pixels with 3 channels and normalization based on ImageNet [22] mean and standard deviation. To quantify the variation between two sets of input images, we computed pairwise differences between their corresponding feature vectors. The feature difference calculation involved element-wise subtraction, resulting in a new set of difference vectors. These vectors captured the underlying dissimilarities between the two sets and served as input for the subsequent classification stage (Fig. 1a).

### Predictive models- classification

Given the limited dataset, it is reasonable to estimate the approximate overlay of tumor ablation coverage in the CT images. The cases were split into four classes of tumor coverage:  ≤ 50%, 50–70%, 70–90%, or ≥ 90%, depicting incomplete, partially incomplete, acceptable, or complete ablation given the technical difficulty of the ablation and potentially desired immune response. The feature difference vectors were fed into a 100-tree random forest model [23], and a prediction of the tumor coverage class was calculated was made by voting (Fig. 1b).

### Predictive models—linear regression

In addition to classification, regression models were built to predict the percentage of tumor coverage. Two regression models were evaluated: a random forest model with 100 trees and a deep neural network (DNN) model. For both models, the input consisted of the previously computed feature difference vectors. The DNN architecture included two sequential hidden layers with 4096 and 1024 nodes, respectively, both using rectified linear unit (ReLU) activation functions. The output layer employed a linear activation function to generate continuous predictions. The DNN model was trained for 30 epochs, and convergence was confirmed. Predictions were limited to ≤ 100% tumor coverage.

### Validation study population

Validation of the study was done on eight patient cases from a separate, single institution. The images were retrospectively collected and analyzed and informed consent was waived by the Institutional Review board. All cases from an Interventional Radiology department treated with RFA between 2001 and 2023 with arterial phase pre-ablation and portal venous phase post-ablation CTs were considered. In line with the training and testing cohort, the pre- and post-treatment images were screened for tumor and ablation zone visibility. The post-ablation images were taken an average of 21 ± 31 days (range 0–90 days) after the procedure. Ground-truth was generated as described earlier by segmentation and elastic registration.

### Statistical methods

To assess the performance of the classification model, accuracy was calculated as the percentage of test cases that were correctly classified and a “one vs. rest” receiver operating characteristic (ROC) analysis was performed. The area under the curve (AUC), true positive rate (TPR or sensitivity), and true negative rate (TNR, or specificity) were calculated. The results are presented as an ROC curve and confusion matrix. All other results are presented in mean ± standard deviation. A Kolmogorov–Smirnov test was used to test for normal distribution of data. A Pearson or Spearman’s Rho correlation analysis was performed to check for association of two variables. To assess the performance of the regression models, the mean square error (MSE), root mean square error (RMSE) and mean average error (MAE) were calculated.

## Results

### Study population

CT images from all 556 patients were screened for sufficient image quality and the presence of a well-defined tumor and ablation zone amenable to segmentation. This resulted in 242 pre-ablation and 338 28-day post-ablation CTs. Among these, 182 matched image pairs (pre- and post-ablation) from the same patients were included in this study.

**Fig. 2:**
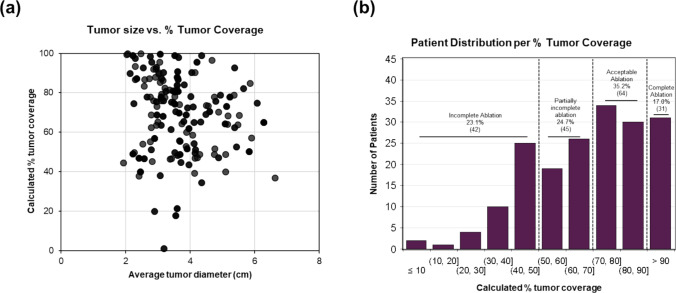
Overall description of patient population included in the study. **a** Comparison of average tumor diameter and percentage of the tumor covered after ablation, generated after ground-truth. **b** Distribution of patient cases based on percentage of the tumor covered after ablation based on ground-truth manual segmentation

The average tumor volume was 31.8 ± 27.6 cm^3^, corresponding to a tumor size of 3.9 ± 3.8 cm assuming a spherical tumor. The average clinically measured tumor diameter on axial 3D imaging was 4.1 ± 1.0 cm. The average ablation volume was 49.3 ± 29.8 cm^3^, corresponding to a zone size of 4.5 ± 3.8 cm assuming a spherical ablation zone. The clinically ablation diameter was not documented. The average percentage of tumor coverage was 68.3 ± 21.0% (Fig. 2a).

For the classification model, the number of (un-augmented) study cases in each of the four classes for the classification model was: *n* = 42 (tumor coverage ≤ 50%), *n* = 45 (tumor coverage = 50–70%), *n* = 64 (tumor coverage = 70–90%), and *n* = 31 (tumor coverage ≥ 90%), or incomplete, partially incomplete, acceptable, or complete ablation (Fig. 2b).

At the various levels of data augmentation resulted in training/testing cohorts consisted of 146:36 image pairs for the original data, 292:72 image pairs for the original and noised data, and 584:144 image pairs for the original and noised data crossed.

### Classification analysis

**Fig. 3:**
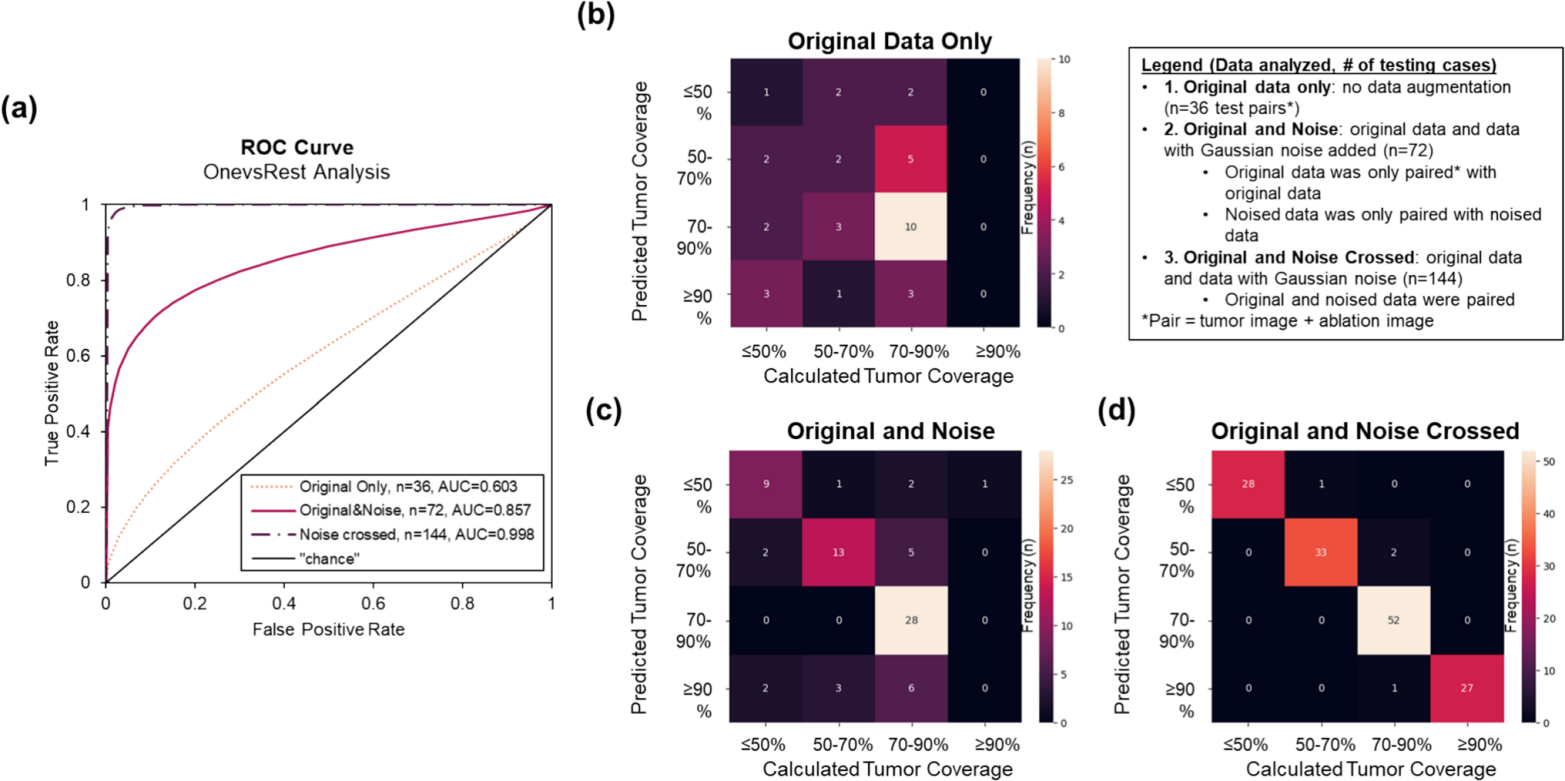
Testing results of the classification models with increasing data augmentation. **a** ROC curve shows the model with the highest data augmentation is likely overfit (AUC = 0.998) suggesting the use of the second model for validation purposes (AUC = 0.857). **b** The data what included original data only had a poor overall performance (AUC = 0.603), compared to the **c** moderately augmented dataset, and the **d** overfit dataset

For classification models, or estimation of tumor coverage, the overall accuracy of the three sets data with increasing data augmentation was 36%, 69%, and 97%. The average AUCs were 0.60, 0.86, and 0.998, TPRs were 0.27, 0.59, and 0.97, and TNRs were 0.77, 0.89, and 0.99, for the first, second, and third cohorts, respectively, that represent increasing data augmentation (Fig. 3). The third cohort appeared overfit, and the AUC of the first cohort was inadequate; therefore, the second cohort was believed to be the best for the validation study population.

### Linear regression analysis

Table 1 summarizes the linear regression models’ performances. This analysis found a slightly higher performance of the random forest model (100-tree) compared to the DNN model (30 epoch). Both models performed best with data augmentation when the original and noised image pairs were crossed or used together.

**Table 1:** Linear regression model results

Linear regression model performance				
			Error (%)	
	Model (number of image pairs, tumor + ablation zone)		RF	DNN
Increasing data augmentation (1–3)	Original data (*n* = 182)	MSE	320.7	2544.4
		RMSE	17.9	50.4
		MAE	15.3	45.2
	Original & noised (*n* = 370)	MSE	253.7	1295.4
		RMSE	15.9	36.0
		MAE	13.1	30.8
	Original & noised crossed (*n* = 728)	MSE	158.6	347.9
		RMSE	12.6	14.2
		MAE	9.8	18.7

RF = random forest (100 trees), DNN = deep neural network (30 epoch), MSE = mean square error, RSME = root mean square error, MAE = mean average error

**Fig. 4:**
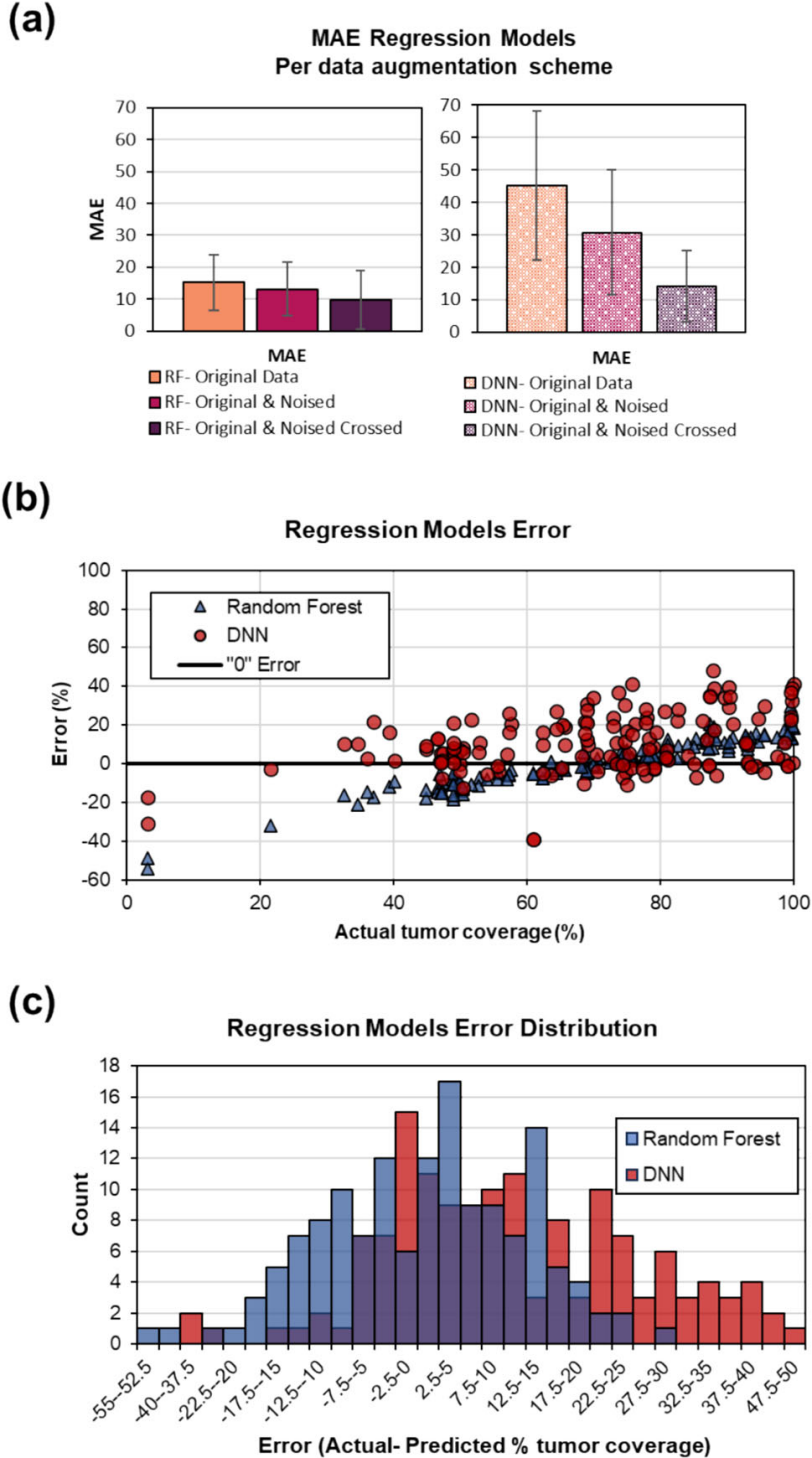
Results of regression model analysis to predict the percentage of the tumor covered. **a** Mean average error (MAE) of three models with increasing data augmentation. The lowest MAE occurred with the highest level of data augmentation using a random forest (RF) model compared to a deep neural network (DNN). **b** Percentage error of the RF and DNN regression models showing the lowest error occurring between 60 and 85% tumor coverage. **c** Distribution of model error

For the best 100-tree random forest model, the error was MSE = 159%, RMSE = 12.6%, and MAE = 9.8% (Fig. 4a). Error was minimal between percentage tumor coverage of 60% and 85% (Fig. 3b) (*r*_p_ = 0.957, *p* < 0.01). At lower tumor coverages, the model overestimated coverage, and at higher tumor coverages, the model underestimated the coverage.

For the best DNN model, the error was MSE = 348%, RMSE = 14.2%, and MAE = 18.7%, showing lower performance to the random forest model. Unlike the random forest model, there was a weak association of error distribution to percent tumor coverage was present (Fig. 4b) (*r*_p_ = 0.363, *p* < 0.01).

**Fig. 5:**
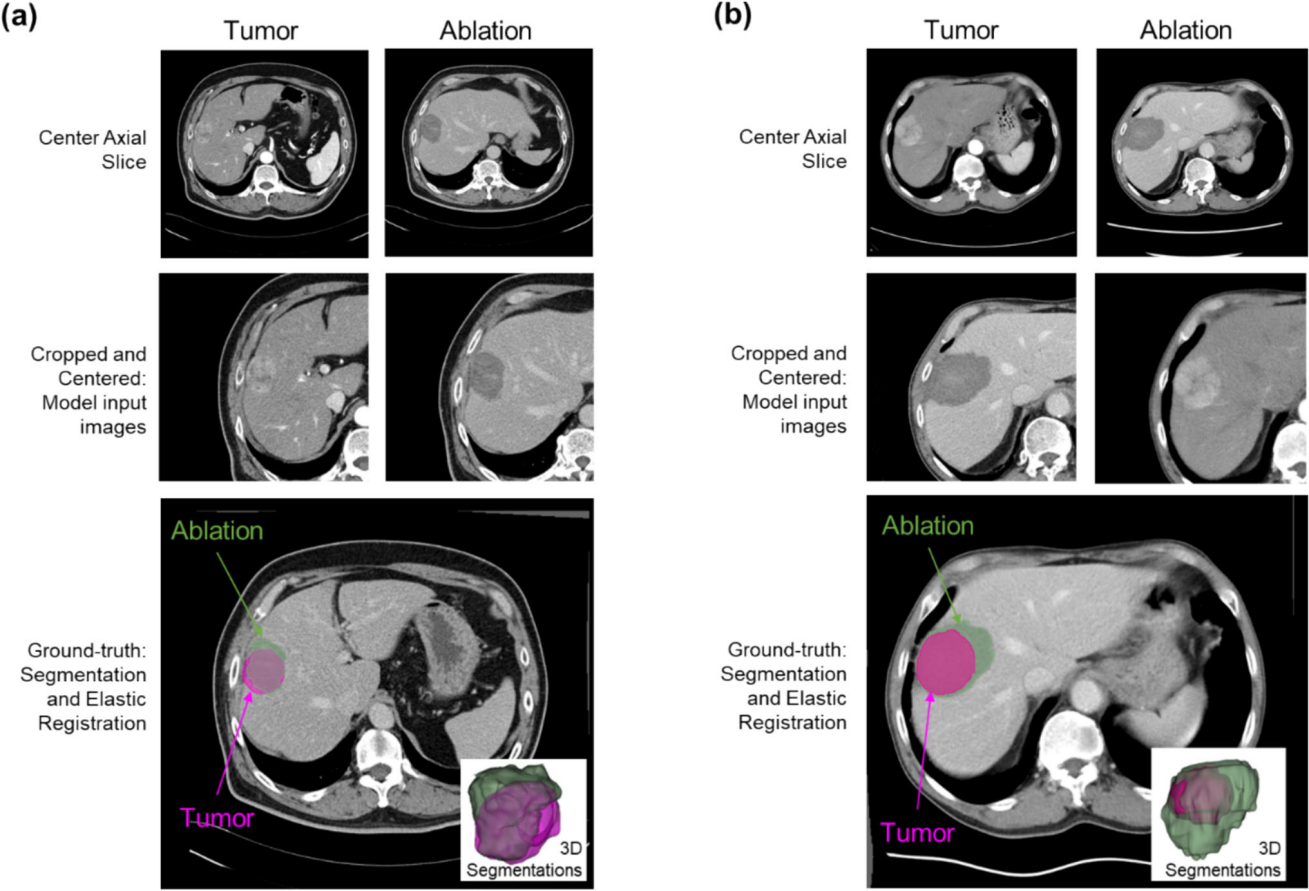
Two example patient cases of the testing cohort showing the ability of classification and regression models in ablation–tumor coverage prediction. **a** A 4.3 cm tumor with a calculated tumor coverage after RFA of 65%. The logistic regression model predicted a tumor coverage of 73% and the classification model correctly predicted moderate coverage of 50–70%. **b** A 4.3 cm tumor with a calculated tumor coverage after RFA of 99%. The logistic regression model predicted a tumor coverage of 76% and the classification model incorrectly predicted good coverage of 70–90%, rather than excellent tumor coverage of > 90%

For both models the error was normally distributed around MAE (Fig. 4c, p = 0.497 and *p* = 0.327 for random forest and DNN models, respectively), and there was a poor association between error and tumor diameter (*r*_s_ =  − 0.426, *p* = 0 and *r*_s_ =  − 0.102, *p* = 0.225 for random forest and DNN models, respectively). The 100-tree random forest and the 30 epoch DNN models were used for testing in the validation cohort. Two representative cases from the testing cohort are shown in Fig. 5 that show the ability of the regression and classification models to predict tumor coverage in the training set.

### Validation study population

From a separate, single, interventional radiology department, eight patients with nine tumors and RF ablation treatments were identified and included in the validation cohort. The post-ablation images were taken an average of 21 ± 31 days (range 0–90 days) after the procedure. The average tumor volume was 8.6 ± 10.9 cm^3^, and the average ablation zone volume was 41.6 ± 34.4 cm^3^. From ground-truth segmentation and elastic registration, the average percentage of the tumor coverage was 60.8 ± 33.5%.

For the classification models, 2/9 (22%), 1/9 (11%), and 2/9 (22%) cases were correctly classified for the models with original data alone, for each of the three models with increasing data augmentation, respectively.

**Fig. 6:**
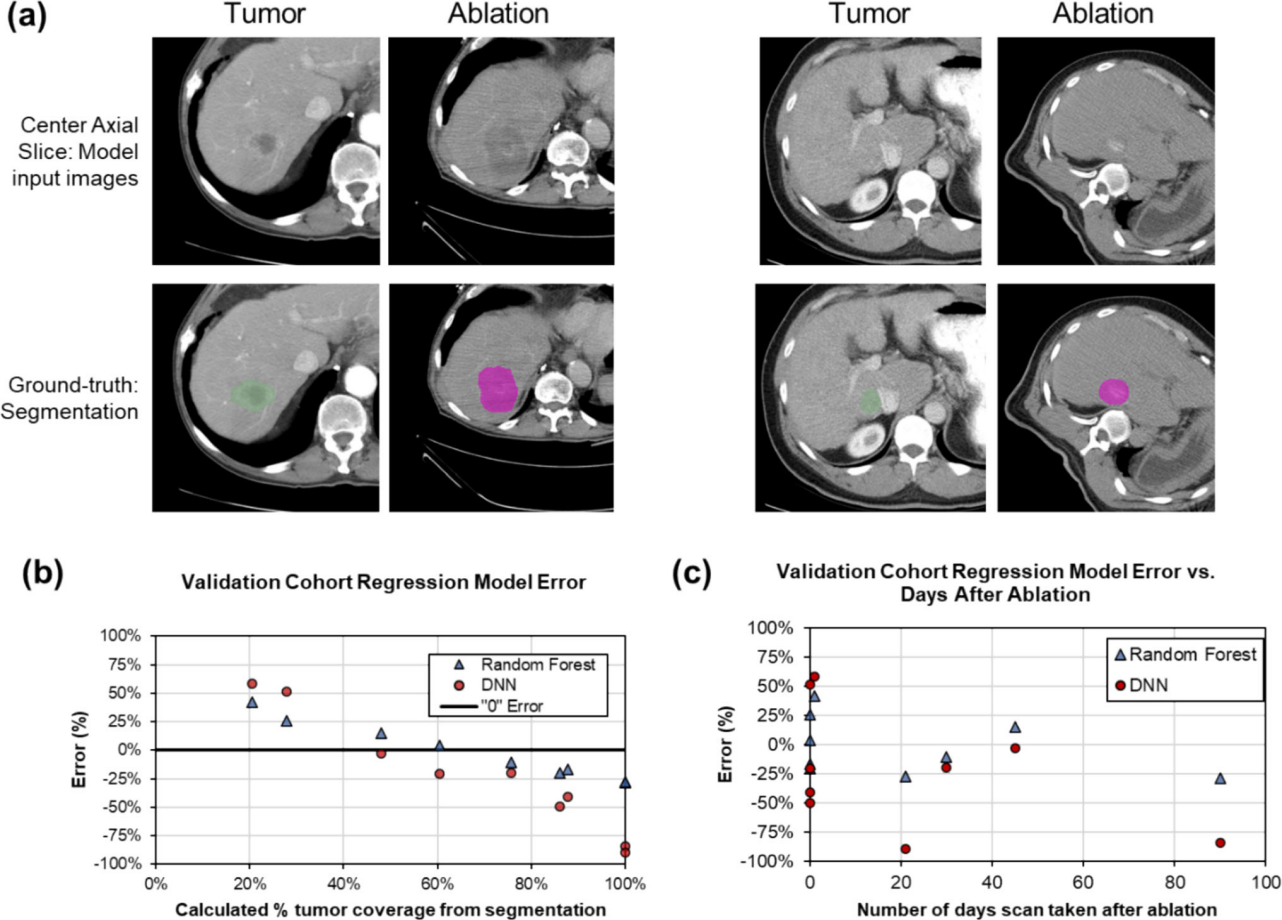
Results of validation of an independent cohort from a single institution. **a** Two example patient cases. Left—a 2.0 cm tumor with calculated 61% tumor overlap. The regression model estimated a tumor coverage of 64% and the classification model incorrectly predicted good coverage of 70–90%, rather than moderate tumor coverage of 50–70%. Right—a 1.5 cm tumor with a calculated tumor coverage of 28%. The regression model estimated a tumor coverage of 54% and the classification model incorrectly predicted good coverage of 70–90%, rather than poor tumor coverage of < 50%. **b** The association of the regression model error and calculated % tumor coverage. Under 60% tumor coverage, both models tended to overestimate tumor coverage. Over 60% tumor coverage, both models tended to underestimate tumor coverage. **c** There was no apparent association between regression model error and the number of days the post-ablation image was taken

In the regression models, MAE was 24% and 46% for the random forest model and the DNN model, respectively. For both models, there was a negative correlation between percent tumor coverage and error (*r*_p_ =  − 0.993, *p* < 0.01 and *r*_p_ =  − 0.976, *p* < 0.01 for the RF and DNN models, respectively). The lowest error appeared around 60% tumor coverage, with lower tumor coverage being overestimated and higher tumor coverage being underestimated (Fig. 6b). There was no apparent association between time of post-ablation imaging and error (Fig. 6c, *r*_p_ =  − 0.391, *p* = 0.299 and *r*_p_ =  − 0.469, *p* = 0.203 for the RF and DNN models, respectively), a positive correlation between tumor diameter and error for the random forest model (*r*_p_ = 0.992, *p* < 0.01), but no correlation between tumor diameter and the DNN model (*r*_p_ = 0.205, *p* = 0.597).

## Discussion

Quantitative and intraprocedural assessment of the technical success of RF ablation could improve overall delivery of the treatment, improve standardization and may improve overall patient outcomes. The adoption of tools that segment and register tumors and ablation zones interprocedurally has been slow but could yield such improvements. The adoption is hampered by the lack of efficient tools and computing power to perform 3D segmentation and registration. This study analyzes the ability to predict technical ablation success by using a single axial image slice at the center of tumor and ablation zone.

The risk of recurrence after ablation of HCC lesions is high, especially for larger tumors, making resection potentially less effective for these larger lesions [3]. Local recurrence risk following percutaneous thermal ablation is related to both nodule dimension and ablation margin [24]. The higher risk of recurrence for larger tumors is likely due to the presence of viable tumor cells beyond the imaged margins of the ablation zone, with resulting incomplete coverage [25, 26].

This study has shown an adequate performance of regression models, which attempt to predict the actual percentage of the tumor covered by the ablation zone. The improved performance of a classification model, which shows varying levels of coverage from inadequate (< 50% coverage) to fully covered tumors, may not be surprising as it is a less discrete prediction compared to the regression model. It was hypothesized that the extraction and comparison of features in 2D would provide sufficient information to predict whether the isocenter of the tumor and ablation zone were in the same axial plane. This study surmised that if the features in the two images were similar, then, by comparing the two images in the Siamese network, there would be a sufficient difference between cases with good and poor overlap. This assumes that for good overlap, the isocenter of the tumor and ablation zone is in a similar axial plane and features such as boney features (ribs or vertebrate) or edges (liver or diaphragm) were similar and would be subtracted from each other. Vastly different axial planes would be reflective of poor overlap, different features, and features that would not be subtracted from each other.

Although this approach can be a fast and efficient approach, a limitation of this approach is that it does not reflect non-symmetric or elongated tumors or ablation zones especially in the cranial-caudal direction. Prior studies have shown that patient with elongated or flatter tumors were more likely to have local recurrence [18]. To this end, significant effort has been put forth toward validating the ability of 3D ablation confirmation tools [7, 8, 27, 28]. Intraprocedural software that evaluates ablation margins in 3D has been under evaluation in ongoing prospective and multicenter trials [10, 29, 30] Earlier studies have even shown the value of 3D ablation zone assessments over 2D manual assessment [5, 6, 31], yet no such tools have been uniformly adopted clinically or mandated as a part of routine clinical care. This may be because tools that incorporates tumor and ablation zone segmentation and elastic registration in real-time have may lack external validation or be cumbersome to use.

As this was a secondary analysis on an existing dataset, and the collection of the data was not designed specifically for the present study, there are additional inherent limitations. First, this was collected as a part of a randomized control trial, where patients received either RFA alone or RFA + thermodox. Although there has been shown to have no impact on survival or progression, it is unknown whether the inclusion of drug has implications on imaging features or tumor coverage. Additionally, post-ablation images were taken approximately 1 month after the treatment. Prior studies have also indicated that post-ablation images taken earlier than 28 days have better ability to predict [32–34]. Zirakchian Zadeh et al. found that intraprocedural evaluation of ablation zone was a better predictor of local tumor progression compared to 4–8 weeks post-ablation [32]. Additionally, CTs taken within 7 days of RF ablation showed a margin of less than 5 mm could predict local progression and overall recurrence [33].

This study has a relatively low average percentage of tumor coverage reported in this study (68.3 ± 21.0%) could be for a variety of factors including the time frame that the post-ablation images were taken (28 days), which would allow for shrinkage and changing liver morphology. Shrinkage has been reported to change the ablated area up to 50% along one diameter [35] and could have led to slight misregistration.

Other limitations include the relatively limited number of patients included in this study (182 patient images, augmented to become 728 image pairs). Prior studies have assessed the quality of the elastic registration; however, elastic deformation was not performed in the comparison of tumor or ablation zone images, which could impact the quality. Patients may have had different respiratory phases or slight variations in body habitus over the 28 days could impact the direct comparison of the images. Additionally, because this was a multinational, multi-institutional study, CT scanning protocols and some ablation parameters and tools may not be uniform between centers. As is suggested in the validation data set (Fig. 6a), variations in the body position between the two scans greatly impacts the ability to implement this tool. The models in the present study also use manual 3D segmentation to identify the center of the tumor or ablation zone. The ability of users to manually select the center of each ROI and the impact on model performance should be investigated. Next, the validation cohort is small and consists of tumors with significantly smaller tumor volumes compared to the training dataset. This could hamper the translation of the models and have an impact on the validation results.

## Conclusions

A model that uses 2D images at the center of the tumor and 1 month post-ablation can accurately estimate ablation tumor coverage for immediate estimation of technical RFA success in testing cohorts. It has limited success in validation cohorts underscoring the need for large, clinically relevant training studies. Clinical tools for ablation confirmation need to be efficient to hope for adoption and treatment optimization. This study suggests that models could achieve ablation confirmation without applying resource-intensive segmentation and registration models.

## Data Availability

The views, information or content, and conclusions presented do not necessarily represent the official position or policy of, nor should any official endorsement be inferred on the part of, the Clinical Center, the National Institutes of Health, or the Department of Health and Human Services. Opinions expressed are those of the authors, not necessarily the NIH. The mention of commercial products, their source, or their use in connection with material reported herein is not to be construed as an actual or implied endorsement by the United States government.
